# The Death of Sir Francis Burdett

**Published:** 1844-04-06

**Authors:** 


### THE DEATH OF SIR FRANCIS BURDETT.

On the 8th of October, 1843, Sir F. Burdett placed himself under the professional care of an hydropathist, who has an establishment in the neighbourhood of London. He was confidently assured that if he adopted the "water-cure" he would have no return of the gout, and, in fact, that all tendency to the disease would be removed. Sir Francis, accordingly, subjected himself to the treatment. Contrary, however, to the predictions of his medical adviser, early in the December following he had a return of his old enemy. At this period Lady Burdett became seriously ill, and Sir Francis was compelled to leave the hydropathic establishment. He, however, frequently visited the institution, and continued to use the cold-water remedies, both at the establishment and at his own house. Lady Burdett (who, although strongly recommended, was not permitted to adopt the hydropathic mode of cure?) died of scirrhus of the stomach. She was attended by Dr. Ferguson and Sir B. Brodie. Sir Francis (so great was his faith in the plan he was pursuing) persisted, almost to the very last, in maintaining that had Lady Burdett submitted to the cold-water treatment, her life would have been prolonged. On the very day upon which Lady Burdett was to have been buried, Sir Francis was seized with an attack of hæmorrhage from the lungs. Up to the Saturday previously to Sir Francis's death the hydropathist was in attendance, but at that time Miss Burdett Coutts peremptorily refused to allow any more hydropathic experiments to be tried upon her father without the full concurrence of his physician. Shortly subsequent to this, a physician in the metropolis, who had frequently been in attendance upon Sir Francis, received a communication from Miss Burdett Coutts, in which she said that she had no hesitation in asserting that the cold-water treatment had destroyed one of the noblest constitutions ever given to man; that it had reduced Sir Francis to a state of great debility, from which it was impossible for him to recover. She also expressed her determination to resist the further use of such quackeries, unless sanctioned by the medical gentlemen who had been consulted. The proximate cause of Sir F. Burdett's death is said to have been an affection of the lungs, and subsequently of the brain, arising, as it is stated, from a translation of the gout from the extremities to the above-named vital organs. For this attack he was attended by Dr. Farr. Sir F. Burdett was quite delirious for at least twenty-four hours preceding his dissolution, and recognised no person near his death-bed. It has been said, that so wedded was Sir Francis to the unfortunate cold-water delusion, that he was in the habit of riding out on horseback, enveloped in wet towels. He thought highly of hydropathy, but he had extended the same confidence previously to St. John Long's mode of treatment, as well as to homœopathy.—*Times.*

